# Hematologic markers and machine learning in predicting placenta accreta: A case–control study

**DOI:** 10.1002/ijgo.70782

**Published:** 2026-02-02

**Authors:** Michael D. Jochum, Kelly D. Albrecht, Yamely Mendez Martinez, Victoria Zhang, Sanmay Sarada, Brian Burnett, Christina C. Reed, Karin A. Fox, Amir A. Shamshirsaz, Michael A. Belfort, Jessian L. Munoz, Hennie A. Lombaard

**Affiliations:** ^1^ Division of Maternal Fetal Medicine, Department of Obstetrics and Gynecology Baylor College of Medicine and Texas Children's Hospital Houston Texas USA; ^2^ Division of Maternal Fetal Medicine, Department of Obstetrics and Gynecology University of Texas Medical Branch Galveston Texas USA

**Keywords:** antenatal diagnosis, hematologic markers, hemorrhage, machine learning, placenta accreta spectrum, quantitative blood loss, ultrasound imaging

## Abstract

**Objective:**

This study aims to enhance antenatal detection of placenta accreta spectrum (PAS) and predict severe hemorrhage at delivery using machine learning by evaluating the association between antenatal hematologic index trends across trimesters, imaging markers, and patient history.

**Methods:**

We retrospectively analyzed 2017–2023 data from a PAS referral center, including demographics, laboratory results, ultrasounds, and outcomes. Patients with confirmed PAS (cases) were compared to those with antenatal risk but no histopathologic evidence of PAS (controls). Statistical analyses and machine learning models were developed to predict PAS. We also used machine learning to predict severe hemorrhage (>1500 mL) in the cases.

**Results:**

A total of 186 PAS cases and 217 controls were identified, showing significant differences in body mass index, gravidity, parity, prior cesarean deliveries, gestational age at delivery, and PAS ultrasound findings. Logistic regression highlighted prior cesarean deliveries (odds ratio [OR] 1.8; 95% confidence interval [CI] 1.3–2.4) and second (OR 28.1; 95% CI 12.7–60.8) or third trimester ultrasound markers (OR 27.6; 95% CI 13.2–61.1) as strong predictors of PAS. Third trimester mean platelet volume was inversely associated with PAS (OR 0.55; 95% CI 0.39–0.78). Machine learning models achieved high accuracy. Model 1 predicted PAS with 90% accuracy. Model 2 predicted PAS with 88.8% accuracy using early gestational hematologic markers. Model 3 predicted severe hemorrhage (>1500 mL) with 74.3% accuracy.

**Conclusion:**

Machine learning models combining patient history, imaging, and hematologic markers detect PAS and predict hemorrhage with up to 90% accuracy. These tools improve antenatal diagnosis of PAS, which enhances maternal outcomes by enabling early identification and better resource allocation.

## 1 INTRODUCTION

Placenta accreta spectrum (PAS) is a serious pregnancy complication characterized by abnormal placental adherence to the myometrium and strongly associated with hemorrhage and maternal morbidity.^1^, ^2^, ^3^, ^4^ Among women with placenta previa, PAS occurs in a median of 11.10% of cases (interquartile range 7.65%–17.35%).^1^ The incidence of PAS has risen significantly, largely due to increasing cesarean delivery rates, with historical estimates ranging from one in 1250 births in 1980^2^ to one in 333 births in 2000–2010.^3^, ^4^ The risk of PAS‐related complications is highest when PAS is identified only at delivery, often precluding transfer to a specialized center. In such centers, multidisciplinary PAS teams and higher provider expertise have been demonstrated to improve outcomes.^5^, ^6^ However, antenatal detection rates remain as low as 33%–50%^7^, ^8^, ^9^ in population‐based studies due to variation in provider expertise in using ultrasound and magnetic resonance imaging (MRI) to evaluate the placenta.^10^, ^11^

Thus, despite advances in imaging as well as standardization of protocols and imaging definitions, the primary barrier to optimal PAS treatment remains the lack of early detection and referral. Emerging research has begun to harness the power of advanced machine learning (ML) to improve antenatal diagnosis and risk stratification of PAS using techniques ranging from traditional classifiers to deep‐learning frameworks that leverage quantitative imaging features extracted from ultrasound and MRI.^12^, ^13^, ^14^ However, prior studies have been limited by small sample sizes. Additional research suggests that integrating hematological markers with imaging findings might enhance diagnostic accuracy.^15^

Inflammation, a key factor in abnormal placental attachment, is reflected in blood parameters like mean platelet volume (MPV), red cell distribution width (RDW), and platelet‐to‐lymphocyte ratio^16^, ^17^ Specifically, because MPV is a marker of platelet activation,^18^ its reduction in PAS cases might reflect an exaggerated local inflammatory response contributing to the disruption of the endometrium–myometrial interface.

We hypothesize that combining imaging, hematological markers, and clinical data through ML can improve PAS prediction and management (FIGO grades 1–3C vs. non‐PAS). This study aims to evaluate PAS detection and predict quantified blood loss (QBL) exceeding 1500 mL based on our institutional cutoff for activating transfusion protocol and the American College of Obstetricians and Gynecologists cutoff for transfusion.

## 2 MATERIALS AND METHODS

### 2.1 Study design

This retrospective case–control study was institutional review board‐approved (Prediction of Placenta Accreta Spectrum Disorders with Antenatal Hematologic Markers, H‐52688) with a waiver of consent from a large academic institution with a multidisciplinary PAS program and 6–20 years of team experience. All patients managed by the multidisciplinary team from January 2017 to May 2023 with clinical and/or histopathology‐proven PAS and singleton pregnancies were included as cases. Patients were excluded if they did not deliver at our location (*n =* 3), had multifetal gestation (*n =* 6), or had hematologic diseases (*n =* 1) that might affect complete blood count (CBC) results (e.g. leukemia). The control group included patients who demonstrated clinical and/or imaging risk factors for PAS but had spontaneous placental separation and no intraoperative findings suggestive of PAS (Figure S1). Additionally, to aid classification, all placentas were submitted to pathology for histopathologic evaluation. The cases involving myometrial fibers or basal plate myometrial fibers observed on the placenta pathology were excluded to minimize bias. Among the controls, two patients required hysterectomy due to significant intraoperative bleeding and clinical suspicion of PAS; however, histopathology ultimately confirmed the absence of PAS. We adhered to the STROBE checklist to ensure that our study design, data collection, and analysis were reported with the utmost transparency and rigor.

### 2.2 Demographic data

**FIGURE 1:**
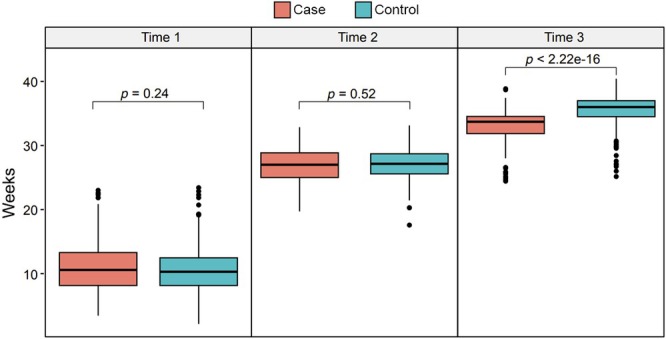
Gestational age at complete blood counts (CBC) collection across trimesters in PAS cases versus controls. Boxplots comparing the distribution of gestational age (weeks) at which CBCs were obtained for placenta accreta spectrum (PAS) cases (salmon) and controls (teal) at three predefined timepoints corresponding roughly to the first (Time 1), second (Time 2), and third (Time 3) trimesters. Wilcoxon rank‐sum tests indicate no significant difference in sampling timing during the first (*P* = 0.24) or second trimester (*P* = 0.52), whereas third‐trimester CBCs were drawn significantly earlier in cases than controls (*P* < 0.001), reflecting earlier delivery in the PAS cohort.

Data that were collected from *n =* 403 patients and coded during chart review included maternal demographic information (Table 1), medical comorbidities and CBC index (Table 2) results from the first, second, and third trimesters. Hematologic markers were collected from CBC results from routine blood testing performed in each trimester, where time points t1, t2, and t3 refer to each trimester. All participants had at least two completed CBC timepoints (Figure 1). Presence or absence of any single ultrasound marker of PAS was treated as a binary predictor. Ultrasound markers of PAS included the presence of multiple lacunae, loss of the retroplacental clear space, bridging vessels, and/or placental bulge. If any single ultrasound marker was present, this case was categorized as “yes,” whereas the absence of all markers was marked as “no.” FIGO clinical grade had been retrospectively extrapolated and recorded in our database based on the clinical description at surgery, and histopathology reports, for cases delivered prior to 2020.^19^ Thereafter, the FIGO grade was assigned prospectively at the time of delivery.^19^ Blood loss was obtained from the operative procedure note. From 2021 onward, quantified blood loss was routinely used at our center, and prior to that time, visually estimated blood loss was used.

**TABLE 1:** Demographics and outcomes PAS versus no PAS.

Variable	PAS	No PAS	*P*‐value
	(*n =* 186)	(*n =* 217)	
Race			0.280
White	134 (72)	156 (71.9)	
Black	35 (18.8)	33 (15.2)	
Asian or Pacific Islander	12 (6.5)	25 (11.5)	
American Indian/ Alaskan Native	1 (0.5)	1 (0.5)	
Not reported	4 (2.2)	2 (0.9)	
Ethnicity			0.632
Hispanic	74 (39.7)	79 (36.4)	
Not Hispanic	109 (58.6)	136 (62.7)	
Not reported	3 (1.6)	2 (9.2)	
Age (years)	34 [30, 37]	34 [30, 37]	0.496
BMI (kg/m^2^)	31.1 [26.9, 36.9]	28.6 [25.8, 33.1]	**0.005**
Gravidity	4 [3, 5]	3 [2, 4]	**<0.001**
Parity	3 [2, 3]	1 [0, 2]	**<0.001**
Delivery mode			**<0.001**
Vaginal	0 (0.0)	11 (5.1)	
Cesarean section	186 (100.0)	206 (94.9)	
Previa			**<0.001**
Yes	161 (86.6)	151 (69.6)	
No	21 (11.3)	19 (8.8)	
Low lying	4 (2.2)	28 (12.9)	
Vasa	0 (0.0)	1 (0.5)	
Resolved	0 (0.0)	18 (8.3)	
Number of prior CD	2 [1,3]	0 [0,1]	**<0.001**
Active smoker	5 (2.7)	6 (2.8)	1.000
Autoimmune disease	2 (1.1)	5 (2.3)	0.459
Chronic hypertension	10 (5.4)	6 (2.8)	0.279
Antenatal steroids	87 (46.8)	50 (23.0)	**<0.001**
GA at delivery	33.7 [31.6, 34.7]	36.3 [34.4, 37.1]	**<0.001**
US features of PAS	168 (90.8)	36 (16.6)	**<0.001**
Peripartum hysterectomy	173 (93.0)	2 (0.9)	**<0.001**
Blood transfusion	119 (64.0)	41 (18.9)	**<0.001**
QBL (mL)	1500 [9 002 251.5]	863 [7 001 200]	**<0.001**

*Note*: Values are presented as median [25th, 75th percentile], or *n* (column %). Bold *P*‐values indicate statistical significance (*P* < 0.05).

Abbreviations: BMI, body mass index; CD, cesarean delivery; GA, gestational age; PAS, placenta accreta spectrum; QBL, quantitative blood loss; US, ultrasound.

**TABLE 2:** Significant hematologic markers PAS versus no PAS.

Variable	PAS (*n =* 186)	No PAS (*n =* 217)	*P*‐value
Timepoint 1			
Mean platelet volume (fL)	10.0 ± 1.2	10.5 ± 1.1	**<0.001**
Red cell distribution width (%)	13.6 ± 1.5	12.4 ± 1.1	**0.003**
Timepoint 2			
Mean platelet volume (fL)	10.3 ± 1.1	10.4 ± 1.0	0.279
Red cell distribution width (%)	13.8 ± 1.7	13.4 ± 1.2	**0.012**
Timepoint 3			
Hemoglobin (g/dL)	10.9 ± 1.2	11.4 ± 1.2	**<0.001**
Neutrophils (cells/μL)	7.7 ± 2.7	7.2 ± 2.8	**0.053**
Neutrophil to lymphocyte ratio	5.0 ± 3.4	4.4 ± 2.8	**0.036**
Platelet to lymphocyte ratio	143 ± 65.7	130.7 ± 49.7	**0.038**
Mean platelet volume (fL)	10.1 ± 0.9	10.5 ± 1.0	**<0.001**
Red cell distribution width (%)	14.8 ± 2.2	14.2 ± 2.0	**0.005**

*Note*: Hematologic parameters were obtained from routine complete blood counts (CBCs) collected at three predefined gestational timepoints corresponding approximately to the first (Timepoint 1), second (Timepoint 2), and third (Timepoint 3) trimesters. Values are presented as mean ± standard deviation. Bold *P*‐values indicate statistical significance (*P* < 0.05).

Abbreviation: PAS, placenta accreta spectrum.

### 2.3 Data analysis

Demographic data, medical comorbidities, hematologic markers, and outcomes were compared using *χ* ^2^ for categorical variables, the unpaired two‐tailed *t*‐test for numeric variables, and logistic regression (SAS, v. 9.4, SAS Institute, Cary, NC) with significance cutoff at *P* < 0.05.

### 2.4 Machine learning models

Machine learning models were generated using R version 4.3.1 (R Core Team, 2023) to create three models for PAS incorporating clinical history, PAS ultrasound markers, and hematologic markers into each model. Model 1 used a random forest model (case vs. control) using indices from blood analyses from all three available timepoints. Model 2 was developed in the same manner but used blood indices only from time points 1 and 2 (first and second trimesters) to test the ability to identify PAS earlier antenatally. Model 3 (hemorrhage risk prediction model) used a gradient‐boosted trees approach, focusing on accurately predicting hemorrhage defined as >1500 mL blood loss accurately in PAS cases.

Training of models 1 and 2 used 80% of the entire dataset, and testing was completed with 20% of the data equally weighted for cases and control. We used linear regression for the prediction of slope for hematologic markers over the available time points for models 1 and 2. Any missing values were imputed using the bagging imputation method. Model training involved 10‐fold cross‐validation repeated five times, and a random search approach was used for hyperparameter tuning to optimize model performance.

Training of model 3 utilized only the PAS patient cohort (*n =* 186), which was split into training (*n =* 151) and testing (*n =* 35) datasets with an 80:20 ratio weighted by blood loss, reflecting a typical split that provides a balance between training complexity and validation accuracy. Model 3 was configured with a Gaussian loss function to directly predict a continuous outcome variable (blood loss in mL). For all models, variable importance scores were calculated to determine the relative weighting of individual features, which significantly affect the model's predictions (Table 3).

**TABLE 3:** Model variable importance scores (scaled).

Description	Variable	Trimester	Model 1 (all timepoints)	Model 2 (no 3rd trimester CBC)	Model 3 (PAS patients only; predicting Qblml)
Gestational age at 3rd CBC	GA3	3rd	*—*	*—*	3.5
Gestational age at delivery	GADEL	3rd	*—*	*—*	12.44
Platelets at 3rd CBC	PLT3	3rd	1.52	*—*	8.53
Mean platelet volume at 3rd CBC	MPV3	3rd	1.83	*—*	4.23
Platelets to lymphocytes ratio at 3rd CBC	PtoLR3	3rd	2.01	*—*	4.36
Neutrophil to Lymphocyte ratio at 3rd CBC	NtoLR3	3rd	1.79	*—*	3.86
Lymphocytes at 3rd CBC	LYMPH3	3rd	1.59	*—*	3.82
Neutrophils at 3rd CBC	NEUT3	3rd	2.17	*—*	2.86
Red Cell Distribution Width at 3rd CBC	RDW3	3rd	1.91	*—*	1.97
Hemoglobin at 3rd CBC	HG3	3rd	1.64	*—*	1.38
Ultrasound features of placenta accreta spectrum	US_PAS	‐	19.48	46.7	0
Number of prior cesarean sections	NopriorCS	‐	10.31	20.87	0.47
Parity	PARITY	‐	4.23	6.29	1.67
Gravidity	GRAV	‐	3.54	3.07	0.9
Gestational age at 2nd CBC	GA2	2nd	2.00	2.97	0.55
Neutrophils at 2nd CBC	NEUT2	2nd	1.74	1.64	2.33
Δ in hemoglobin	slope_HG	‐	1.83	1.58	4.58
Mean platelet volume at 2nd CBC	MPV2	2nd	1.96	1.44	1.24
Δ in platelets	slope_PLT	‐	2.46	1.42	2.01
Δ in mean platelet volume	slope_MPV	‐	2.28	1.41	1.5
Body mass index	BMI	‐	1.80	1.36	2.58
Δ in neutrophils	slope_NEUT	‐	2.53	1.29	4.16
Δ in neutrophil‐to‐lymphocyte ratio	slope_NtoLR	‐	1.88	1.24	1.59
Platelets‐to‐lymphocytes ratio at 2nd CBC	PtoLR2	2nd	1.61	1.04	2.17
Slope of platelets‐to‐lymphocytes ratio	slope_PtoLR	‐	2.93	0.98	1.16
Δ in red cell distribution width	slope_RDW	‐	2.07	0.89	2.39
Hemoglobin at 2nd CBC	HG2	2nd	1.26	0.8	0.78
Gestational age at 1st CBC	GA1	1st	1.64	0.7	2.53
Red cell distribution width at 2nd CBC	RDW2	2nd	1.46	0.66	1.48
Red cell distribution width at 1st CBC	RDW1	1st	2.07	0.62	0.26
Platelets at 1st CBC	PLT1	1st	1.49	0.59	1.33
Δ in lymphocytes	slope_LYMPH	‐	1.69	0.58	1.98
Mean platelet volume at 1st CBC	MPV1	1st	1.68	0.55	1.28
Neutrophil‐to‐lymphocyte ratio at 1st CBC	NtoLR1	1st	1.47	0.38	1.89
Hemoglobin at 1st CBC	HG1	1st	1.22	0.21	0.65
Lymphocytes at 1st CBC	LYMPH1	1st	1.51	0.19	3.57
Neutrophil‐to‐lymphocyte ratio at 2nd CBC	NtoLR2	2nd	1.73	0.19	1.92
Neutrophils at 1st CBC	NEUT1	1st	1.54	0.13	1.52
Lymphocytes at 2nd CBC	LYMPH2	2nd	1.27	0.12	1.66
Platelets‐to‐lymphocytes ratio at 1st CBC	PtoLR1	1st	1.54	0.09	0.57
Platelets at 2nd CBC	PLT2	2nd	1.30	0	2.33

*Note*: Variable importance scores demonstrating the relative contribution of each feature to model performance. Each variable is scaled within each model with higher values indicating a greater influence on prediction.

Abbreviation: BMI, Maternal body mass index calculated at the first prenatal visit (kg/m²) ; CBC, complete blood count; GA1, Gestational age (in weeks) at the time of the first‐trimester complete blood count (CBC) ; GA2, Gestational age (in weeks) at the time of the second‐trimester CBC ; GA3, Gestational age (in weeks) at the time of the third‐trimester CBC ; GADEL, Gestational age (in weeks) at delivery ; GRAV, Total number of prior pregnancies, including current pregnancy ; HG1 / HG2 / HG3, Hemoglobin concentration measured at the first, second, and third trimester CBC, respectively; LYMPH1 / LYMPH2 / LYMPH3, Absolute lymphocyte count measured at the first, second, and third trimester CBC, respectively ; MPV1 / MPV2 / MPV3, Mean platelet volume measured at the first, second, and third trimester CBC, respectively ; RDW1 / RDW2 / RDW3, Red cell distribution width measured at the first, second, and third trimester CBC, respectively ; NEUT1 / NEUT2 / NEUT3, Absolute neutrophil count measured at the first, second, and third trimester CBC, respectively ; NopriorCS, Number of prior cesarean deliveries NtoLR1 / NtoLR2 / NtoLR3, Neutrophil‐to‐lymphocyte ratio calculated from CBC values at the first, second, and third trimester, respectively ; PLT1 / PLT2 / PLT3, Platelet count measured at the first, second, and third trimester CBC, respectively ; PtoLR1 / PtoLR2 / PtoLR3, Platelet‐to‐lymphocyte ratio calculated from CBC values at the first, second, and third trimester, respectively ; PARITY, Number of prior pregnancies reaching viable gestational age ; Qblml, Quantitative blood loss at delivery, measured in milliliters ; slope_HG, Change in hemoglobin concentration across pregnancy, modeled as the longitudinal slope between CBC timepoints ; slope_PLT, Change in platelet count across pregnancy ; slope_MPV, Change in mean platelet volume across pregnancy ; slope_NEUT, Change in absolute neutrophil count across pregnancy ; slope_LYMPH, Change in absolute lymphocyte count across pregnancy ; slope_RDW, Change in red cell distribution width across pregnancy ; slope_NtoLR, Change in neutrophil‐to‐lymphocyte ratio across pregnancy ; slope_PtoLR, Change in platelet‐to‐lymphocyte ratio across pregnancy ; US_PAS, Presence of ultrasound features suggestive of placenta accreta spectrum, based on standardized antenatal ultrasound criteria (binary variable).

## 3 RESULTS

Throughout the study period, we identified 186 cases (PAS) and 217 controls (no PAS) (Figure S1), with no differences in age or self‐reported race and ethnicity (Table 1). There were significant differences in median body mass index, gravidity, parity, number of prior cesarean deliveries, gestational age at delivery and presence of ultrasound findings of PAS between cases and controls (Table 1). Logistic regression confirmed that increasing number of prior cesarean deliveries remained a significant predictor of presence of PAS (odds ratio [OR] 1.8; 95% confidence interval [CI] 1.3–2.4). The presence of ultrasound markers for placenta accreta demonstrated the most significant independent effect on detection of PAS in both the second (OR 28.1; 95% CI 12.7–60.8) and third trimesters (OR 27.6; 95% CI 13.2–61.1). MPV measured in the third trimester was inversely associated with PAS, with each unit increase in MPV corresponding to a 45% reduction in the odds of PAS (OR 0.55; 95% CI 0.39–0.78) (Table 2).

### 3.1 Supervised machine learning model 1 predicts placenta accreta spectrum with 90% accuracy

**FIGURE 2:**
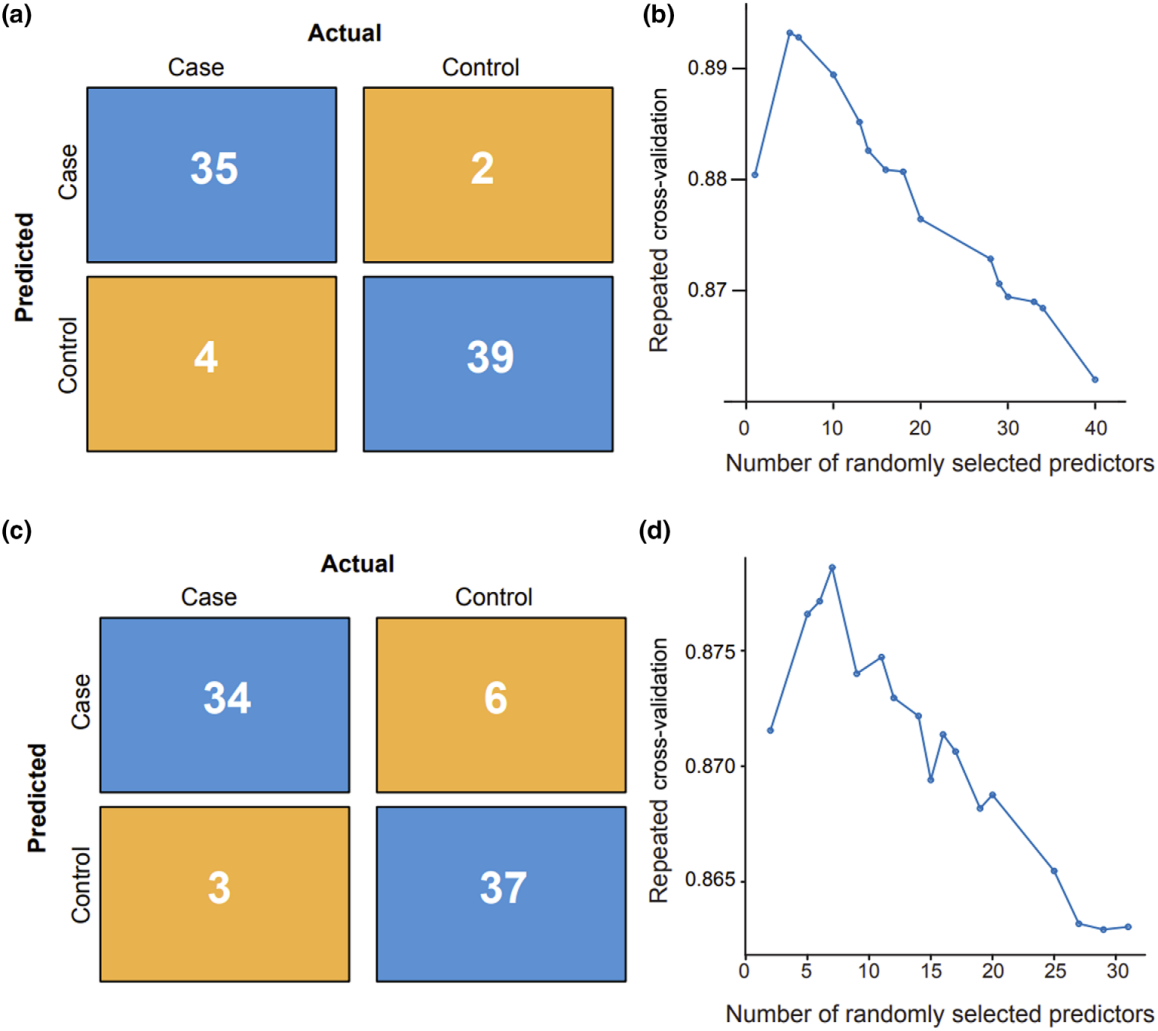
Performance of antenatal PAS prediction models. (a) Confusion matrix for model 1 (random forest using complete blood count (CBC) indices from all three trimesters plus clinical and ultrasound features), showing 34 true positives, five false positives, three false negatives, and 38 true negatives for antenatal placenta accreta spectrum (PAS) detection (overall accuracy 90%). (b) Repeated 10‐fold cross‐validation accuracy for model 1 as a function of the number of randomly selected predictors, peaking at 11 variables before declining. (c) Confusion matrix for model 2 (random forest using only first‐ and second‐trimester CBCs with clinical and ultrasound data), showing 34 true positives, six false positives, three false negatives, and 37 true negatives (accuracy 88.8%). (d) Cross‐validation accuracy for model 2 across varying numbers of predictors, with maximal performance at eight to 10 variables before a gradual decrease.

As PAS can be nuanced and difficult to diagnose solely on ultrasound,^20^ there is a critical need for improved and accessible diagnostic methods. To address this gap, we constructed a ML random forest classification model using the complete dataset (see methods section) including patient demographics, PAS ultrasound findings classified as yes/no, and hematologic markers aimed at enhancing the antenatal detection of PAS. This model achieved a high accuracy of 92.5% (95% CI: 84.39%–97.2%) in identifying PAS cases (Figure 2a) with a sensitivity of 89.74%, specificity of 95.12%, positive predictive value (PPV) of 94.6%, and negative predictive value (NPV) of 90.70% for antenatal PAS detection. Interestingly, the model achieved maximal accuracy using only nine of the input variables (Figure 2b).

Variable importance scores for model 1 (Table 3) indicated that timepoint 3 CBC measurements including the rate of change in hematologic markers, specifically in RDW and MPV, significantly contributed to the accuracy of PAS detection in the model. However, as these data were obtained upon admission to labor and delivery, at which point it is often too late to facilitate the timely transfer of patients from rural areas to specialized centers, we next sought to evaluate the potential of using only earlier pregnancy time points for PAS detection.

### 3.2 Machine learning model 2 using only hematological markers at two early timepoints predicts placenta accreta spectrum with 88.8% accuracy

Aiming to detect PAS earlier in pregnancy, we first analyzed the rate of change in complete blood count (CBC) indices using only first and second trimester CBC results. We then re‐ran the ML prediction model (model 2) utilizing these two timepoints. Model 2 demonstrated an accuracy of 88.8% (95% CI: 79.7%–94.7%) in predicting PAS during the second trimester (Figure 2c). The model achieved a sensitivity of 91.89%, specificity of 92.5%, positive predictive value (PPV) of 91.2%, and NPV of 86.1%. Interestingly, the model achieved the maximal receiver operator curve using only seven of the randomly selected predictors (Figure 2d), including gestational age and neutrophils at time point 2, and the rate of change in hemoglobin rose in importance (Table 3). These performance metrics indicate that even using only the first and second trimester hematologic markers, combined with patient history and ultrasound findings, maintains high accuracy in early gestational prediction of PAS.

### 3.3 Machine learning model 3 can predict severe hemorrhage among placenta accreta spectrum patients with 74% accuracy

The severity of PAS varies widely, with some patients experiencing severe hemorrhage, necessitating advanced interventions such as the use of intra‐aortic balloon occlusion (Control of Bleeding, Resuscitation, Arterial Occlusion System, COBRA).^21^ Identifying these patients before delivery is crucial for activating appropriate resources to prevent severe outcomes. Yet, there are no models capable of predicting which cases are likely to experience significant blood loss (>1500 mL) To address the need for predicting hemorrhage in PAS patients, we used supervised ML to predict which women with PAS would have severe hemorrhage (QBL >1500 mL). This model demonstrated moderate accuracy of 74.3% (95% CI: 56.7%–87.5%), significantly better than the baseline (no information rate) of 54.29% (*P* = 0.012). The most significant predictors were gestational age at delivery, ultrasound findings for PAS, and rate of change in hematologic indices like MPV and RDW (Table 3). Thus, the model's inclusion of the rate of change in hematologic markers allowed for more accurate predictions of severe hemorrhage (QBL >1500 mL), making it more effective than relying on traditional risk factors alone.

## 4 DISCUSSION

In this retrospective study, we demonstrate that ML models integrating longitudinal hematologic trends, clinical history, and ultrasound findings achieved high accuracy for antenatal detection of PAS and QBL. Using random forest models, we predicted PAS with up to 92.5% accuracy using data from all three trimesters and maintained 88.8% accuracy even when limited to first and second trimester hematologic indices. Finally, we were able to predict hemorrhage with a moderate accuracy of 74.3% among patients with PAS.

A key strength of our ML models is the incorporation of the longitudinal changes in hematologic indices with clinical and imaging risk factors associated with PAS. Prior studies have demonstrated a variable range of diagnostic performance using ultrasound or MRI image classification, reporting area under the curve (AUC) ranging from approximately 0.71 to 0.96, most rely predominantly on a single image time point or late in gestation.^12^ For instance, Wang et al. used deep learning for segmentation and classification of placental MRIs, achieving AUCs of 0.860 and 0.897, respectively, but required specialized imaging, segmentation workflows, and expert interpretation.^22^ Although our models were not specifically designed for image classification, they perform in the upper tier of this range and are competitive with nearly all published studies. There is very limited literature evaluating hematologic markers as predictors of PAS, and those studies have relied almost exclusively on single‐timepoint measurements. The most relevant comparison to our work is the recent diagnostic accuracy study by Al‐Nuaimi and Al‐Nuaimi, which evaluated early pregnancy hematological indices in women at high risk for PAS. In that study, RDW demonstrated the strongest individual predictive performance, with an AUC of 0.707.^23^ While this analysis considered these variables in isolation from a single first trimester time point, our models leverage the longitudinal nature and the complexity of interactions among them. For instance, demographic factors provide important context for a patient's baseline risk profile. When combined with ultrasound findings and the rate of change in hematologic markers reflecting systemic inflammation and hemostatic function, our ML approach yields a more nuanced, accurate, and clinically useful risk‐stratification tool.

Previously, development of PAS was attributed to a primary defect in trophoblast biology, leading to excessive invasion of the myometrium,^24^ whereas recent pathological studies suggest that PAS is not a disorder of invasive placentation but, rather, a disorder primarily resulting from a uterine scar and its effects upon placental tissue developing within it. Scar implantation leads to secondary placental abnormalities.^25^, ^26^ High vascular flow from the placenta into this area of compromised myometrium causes oxidative and shear stress.^27^ Our findings of lower MPV in patients with PAS and associated higher platelet count^28^ suggest activation of the platelet cascade. This might explain the dense fibrinoid deposition in the area of placental adherence that has been identified upon gross and histopathologic examination of PAS.^29^

Given the presence of high‐velocity vascular flow from the placenta, the combination of oxidative stress, shear stress, and platelet activation contributes to excessive fibrinoid deposition at the basal plate, which progresses over time.^25^ The inverse relationship between MPV and PAS in our data suggests that platelet dynamics might contribute to the development of PAS.

Lower MPV has been associated with systemic inflammation.^30^ Thus, the importance of hematological indices associated with inflammation in our data suggests that PAS might be associated with broader, systemic inflammation; however, identifying causation and the temporal relationship between inflammatory state and placental implantation and development will require more targeted research. A deeper understanding of these inflammatory mechanisms could guide the development of interventions aimed at modulating the local and systemic inflammatory environment. Strategies such as optimizing uterine health and minimizing inflammation in women with a history of uterine surgery or other risk factors could help mitigate the risk of PAS in subsequent pregnancies.

The limitations of this study include that we were unable to match cases and controls by the number of cesarean deliveries, although we controlled for this in the logistic regression. We additionally recognize that our control group did not undergo hysterectomy or partial uterine wall resection, as there was no intraoperative concern for PAS. Consequently, although all controls had clinically normal placental separation and no surgical or pathologic findings suggestive of PAS, true histologic exclusion of PAS involving the uterine wall cannot be confirmed. PAS ultrasound findings were listed collectively and in a binary fashion (present/absent), and various combinations of individual ultrasound markers were not studied, which could potentially provide more nuanced detection if integrated. We used an internal test set but have not yet internally validated our findings using prospectively collected data. Our findings might not be generalizable to centers without PAS imaging and surgical experts. Finally, in cases when QBL was not available, we relied on estimated blood loss, which might explain model 3's moderate accuracy.

## 5 CONCLUSION

In conclusion, ML models incorporating patient historical factors, imaging characteristics, and hematologic markers are a novel approach to improve upon current detection accuracy of PAS. Future research incorporating individual and combined ultrasound markers into the ML models (e.g., bridging vessels, placental bulge, and loss of retroplacental clear space) along with established scoring systems (e.g., the placenta accreta index^31^), investigational imaging protocols (ferumoxytol‐enhanced MRI^32^, ^33^), and artificial intelligence ultrasound software could be used to further refine these models and improve detection accuracy. Future research will improve generalizability by expanding internal and external validation with prospective and diverse patient cohorts. Ultimately, these findings could support the development of an online calculator to be used in PAS risk assessment in any outpatient setting.

## AUTHOR CONTRIBUTIONS

Design: K.A., K.F., A.S., J.M., M.B, H.L. Planning: K.A., B.B., K.F., A.A. S., J.M, M.B., H.L. Conduct: K.A., B.B., K.F., M.J., A.A.S., J.M, H.L. Data Analysis: M.J., Y.M., C.R., S.S., V.Z. Manuscript writing: M.J., B.B, K.A., Y.M., V.Z., S.S., C.R., K.F, J.M, A.S., M.B., H.L.

## FUNDING INFORMATION

This project was funding using internal funding on behalf of Texas Children's Research Vision, and internal departmental research funding on behalf of Baylor College of Medicine, Department of Obstetrics and Gynecology. There are no applicable grant numbers for these funding sources.

## CONFLICT OF INTEREST STATEMENT

KAF reports financial support from Elsevier Publishing (Associate Editor, AJOG, Deputy Editor AJOG MFM), Wolters‐Kluwer (UpToDate chapter author), and serves on the boards for the Pan‐American Society for Placenta Accreta Spectrum and International Society for Placenta Accreta Spectrum.

## Supporting information


**Figure S1.** Study flow diagram for cohort selection and case–control classification. Of *n* = 413 patients referred for suspected placenta accreta spectrum (PAS), *n* = 10 were excluded, yielding a final analytic cohort of 217 controls and 186 PAS cases. PAS cases were distributed by severity as FIGO 1 (*n* = 29), FIGO 2 (*n* = 59), and FIGO 3 (*n* = 98).

## Data Availability

The data that support the findings of this study are available on request from the corresponding author. The data are not publicly available due to privacy or ethical restrictions.
