# Supplementary figures and images for: Hematologic markers and machine learning in predicting placenta accreta: A case–control study
Source: Int J Gynaecol Obstet. 2026 Feb 2;173(3):1596–604. doi: 10.1002/ijgo.70782 (PMC13173623; doi:10.1002/ijgo.70782)

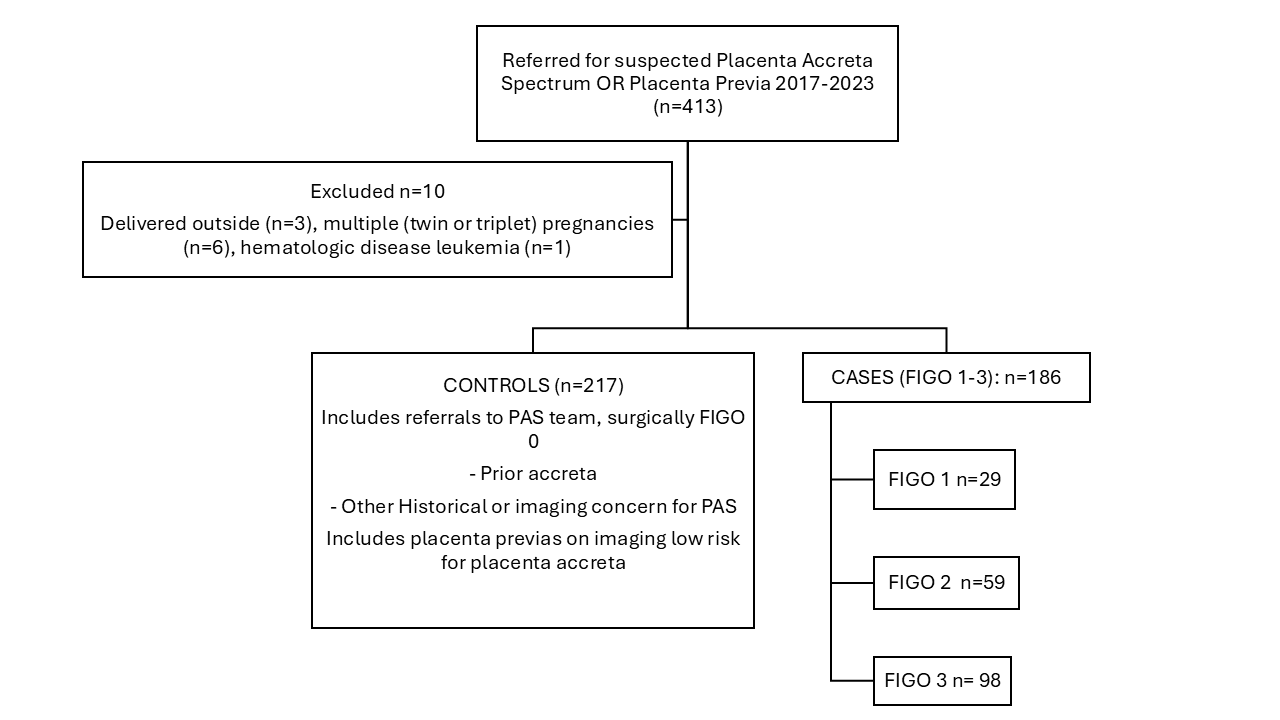

Supplement: Supplementary file 1 — Figure S1. Study flow diagram for cohort selection and case–control classification. Of n = 413 patients referred for suspected placenta accreta spectrum (PAS), n = 10 were excluded, yielding a final analytic cohort of 217 controls and 186 PAS cases. PAS cases were distributed by severity as FIGO 1 (n = 29), FIGO 2 (n = 59), and FIGO 3 (n = 98). [file IJGO-173-1596-s001.png]
